# Zebrafish swimming in the flow: a particle image velocimetry study

**DOI:** 10.7717/peerj.4041

**Published:** 2017-11-14

**Authors:** Violet Mwaffo, Peng Zhang, Sebastián Romero Cruz, Maurizio Porfiri

**Affiliations:** Department of Mechanical and Aerospace Engineering, New York University Tandon School of Engineering, Brooklyn, NY, United States of America

**Keywords:** Flow physics, Zebrafish, Vortex, Strouhal number, PIV, Swimming

## Abstract

Zebrafish is emerging as a species of choice for the study of a number of biomechanics problems, including balance development, schooling, and neuromuscular transmission. The precise quantification of the flow physics around swimming zebrafish is critical toward a mechanistic understanding of the complex swimming style of this fresh-water species. Although previous studies have elucidated the vortical structures in the wake of zebrafish swimming in placid water, the flow physics of zebrafish swimming against a water current remains unexplored. In an effort to illuminate zebrafish swimming in a dynamic environment reminiscent of its natural habitat, we experimentally investigated the locomotion and hydrodynamics of a single zebrafish swimming in a miniature water tunnel using particle image velocimetry. Our results on zebrafish locomotion detail the role of flow speed on tail beat undulations, heading direction, and swimming speed. Our findings on zebrafish hydrodynamics offer a precise quantification of vortex shedding during zebrafish swimming and demonstrate that locomotory patterns play a central role on the flow physics. This knowledge may help clarify the evolutionary advantage of burst and cruise swimming movements in zebrafish.

## Introduction

Zebrafish (*Danio rerio*) has emerged as a species of choice for their genetic and neurological similarity to humans, high reproduction rate, and ease of maintenance (Miklósi & Andrew, 2006; Lawrence, 2007; Spence et al., 2008; Brock et al., 2017). Their complex locomotory patterns have been extensively analyzed in biomechanics research, addressing the development of balance (Fuiman & Webb, 1988; Bianco et al., 2012; Ehrlich & Schoppik, 2017), the mechanisms underpinning aggregation into schools (Miller & Gerlai, 2012; Laan, Gil de Sagredo & De Polavieja, 2017), and neuromuscular transmission (Borla et al., 2002; Drapeau et al., 2002; Fetcho, Higashijima & McLean, 2008).

Zebrafish locomotion is often characterized by a so-called burst-and-coast swimming style, in which “fish move forward (burst) in a single motion and glide (coast) to a slow speed, or stop from which they burst forward again” (Kalueff et al., 2013). Alternative to burst-and-coast, larval zebrafish may also display a continuous and steady swimming pattern, in which persistent body undulations are used for cruising (Müller & Van Leeuwen, 2004). Burst-and-coast locomotion is common to a number of species (Rome et al., 1988; Rome, Swank & Corda, 1993; Wardle, Videler & Altringham, 1995; Wakeling, 2001), which may employ this swimming style in addition to cruising (Weihs & Webb, 1983; Rome et al.,  1988).

Particle image velocimetry (PIV) (Raffel et al., 2013) has been proposed as a powerful technique for visualizing the flow physics around swimming fish, toward a mechanistic understanding of fish locomotion, and more recently, a few studies have undertaken indirect pressure reconstruction to further explain fish hydrodynamics (Dabiri et al., 2014; Gemmell et al., 2015). With respect to cruising, vortical structures in the wake of sunfish (Drucker & Lauder, 2000; Lauder & Madden, 2007), eel (Müller et al., 2001; Müller & Van Leeuwen, 2006), and rainbow trout (Blickhan et al., 1992) have all been visualized using planar PIV. In these studies, two patterns of vortices have been observed in the swimming plane of a cruising fish, namely: (i) two rows of isolated vortices with alternating signs located on both sides of the tail trajectory, as seen in rainbow trout (Blickhan et al., 1992); and (ii) two rows of double vortices, as reported for eel (Müller et al., 2001). The hydrodynamics of these two vortex patterns are considerably different, thereby influencing the associated thrust production (Müller et al., 1997; Müller et al., 2001).

The former structure has been hypothesized to be a cross-sectional view of a series of vortices linked together forming a chain (Müller et al., 2001; Müller & Van Leeuwen, 2006), in agreement with recent volumetric PIV on cruising bluegill sunfish and cichlid fish (Flammang et al., 2011), revealing a series of linked vortex rings forming a complex three-dimensional chain. The latter vortex pattern has been proposed to entail the cross-section of two rows of isolated vortex rings distributed on both sides of the tail trajectory (Müller et al., 2001; Müller & Van Leeuwen, 2006). This explanation is supported by early work on the visualization of the flow around cruising pearl danio (*Danio albolineatus*) (Rosen, 1959), a fish species of the *Danio* genus where zebrafish belongs, and more recent planar PIV experiments on zebrafish larvae (Müller, Van den Boogaart & Van Leeuwen, 2008). Through observations at different planes, these studies have demonstrated the existence of two rows of vortex pairs observed in the wake of the cruising fish, which is reminiscent of a double row of vortex rings structure in three dimensions.

With respect to burst-and-coast swimming, PIV experiments were conducted on adult zebrafish in placid water (Müller, Stamhuis & Videler, 2000), showing the presence of two vortices that are shed away from the tail during each tail beat. However, vortex patterns in the wake of cruising adult zebrafish have never been studied, possibly because adult zebrafish adopt burst-and-coast more than cruising in placid water (Müller, Van den Boogaart & Van Leeuwen, 2008). As shown by Budick and O’Malley (Budick & O’Malley, 2000) through the use of automated tracking software, body bending amplitude and frequency, heading direction change, and swimming speed of larval zebrafish varied as a function of their locomotory patterns. Bursting and cruising have been associated with escaping (Maximino et al., 2010) and prey capturing behaviors (Budick & O’Malley, 2000), suggesting that the hydrodynamics elicited by both locomotory patterns should be different.

To the best of our knowledge, little is known about zebrafish swimming against a water current. A study on the natural habitats of zebrafish revealed that zebrafish live indeed in secondary and tertiary channels connected to a main stream/river where the flow speed is in the range of 3.5–13.9 cm/s (Arunachalam et al., 2013). Zebrafish have shown great capability of adjusting their behavior to environmental stimuli (Olszewski et al., 2012; Suriyampola et al., 2016). A recent study has found that zebrafish become more aggressive and form less cohesive groups when they are transitioned from still water to a weak water flow environment (Suriyampola et al., 2016). Therefore, it is tenable to hypothesize that zebrafish may adapt their locomotory patterns in response to a water current.

To fill this knowledge gap, we studied zebrafish swimming in a miniature swim tunnel designed to produce laminar flow. Zebrafish swimming was recorded at different flow speeds, and locomotory indicators, including tail beat amplitude (*A*), tail beat frequency (*f*), swimming speed relative to the flow (*V*), and heading angle (*θ*), were analyzed using an in-house digital image analysis program. One aim of this investigation was to quantify the variation in the tail beat undulations, heading direction, and swimming speed as a function of the flow speed.

To shed light on zebrafish locomotion, we scored the following nondimensional numbers: (i) the Strouhal number (St = *fA*∕*V*), encapsulating the dependence of swimming on tail beating, and (ii) the Reynolds number (Re = *ρVL*∕*μ*, where *ρ* is the fluid density and µ  is the fluid viscosity), summarizing viscous versus inertial effects in swimming. From published data on swimming animals with various body lengths (Gazzola, Argentina & Mahadevan, 2014), it has been demonstrated that in the inertial regime, undulatory gaits lead to Strouhal numbers that are independent of the Reynolds number for Re > 10^4^ and only weakly dependent on Re for Re < 10^4^. Data on swimming across different species indicated that the Strouhal number for cruising concentrates in a narrow range 0.2 < St < 0.4 (Taylor, Nudds & Thomas, 2003). Such a narrow range might correspond to a highly advantageous gait, as shown by measurements of the propulsive efficiency of a flapping body (Taylor, Nudds & Thomas, 2003). Based on this grounding, we hypothesized that when cruising against a water current, zebrafish operate within the same range of St toward attaining a high propulsive efficiency.

In the experiments, we simultaneously analyzed the flow field around the fish using PIV toward an improved understanding of vortical structures in the wake of swimming zebrafish and the concurrent pressure field. From the flow physics, we sought to explore the topology of vortex shedding for different zebrafish locomotory patterns. The strength of the vortices shed during burst and cruise swimming was quantified through the vortex circulation, which is associated with momentum exchange between the fish and the flow. The dependence of circulation on Strouhal number was further examined, under the premise that increasing the tail beat amplitude should produce stronger vortices, that is, larger circulation. Similarly, from the pressure field, we attempted to explain detailed variations in the way zebrafish interact with the surrounding water flow as a function of their locomotory pattern.

This study may provide valuable information on how zebrafish swim in a dynamic flow environment, which may support parallel efforts on the computational modeling of zebrafish swimming and refined experiments to explain the evolutionary advantage of the burst and cruise swimming styles in their natural habitat.

## Materials and Methods

The experimental procedure described in this study was approved by the University Animal Welfare Committee (UAWC) of the New York University under protocol number 13-1424.

### Animals and housing

A total of 12 zebrafish (*Danio rerio*) of wild-type variety, with an average body length of 3.4 ± 0.2 cm, were purchased from an online aquarium vendor (LiveAquaria.com, Rhinelander, WI, USA) for the experiments. Fish were housed in large water tanks of 37.8 L (10 gallons), with a maximum density of one fish per two liters of water, and with temperature and acidity maintained at 26 ±1 °C and 7.2 pH (Abreu et al., 2017; Johnson & Hamilton, 2017), respectively. Prior to the experiments, fish were acclimatized for 12–15 days under a 12 h light/12 h dark photoperiod (Johnson & Hamilton, 2017) and fed with commercial flake food (Nutrafin Max; Hagen Corp., Montreal, Canada) between 6 pm and 7 pm every day.

### Miniature swim tunnel

**Figure 1:**
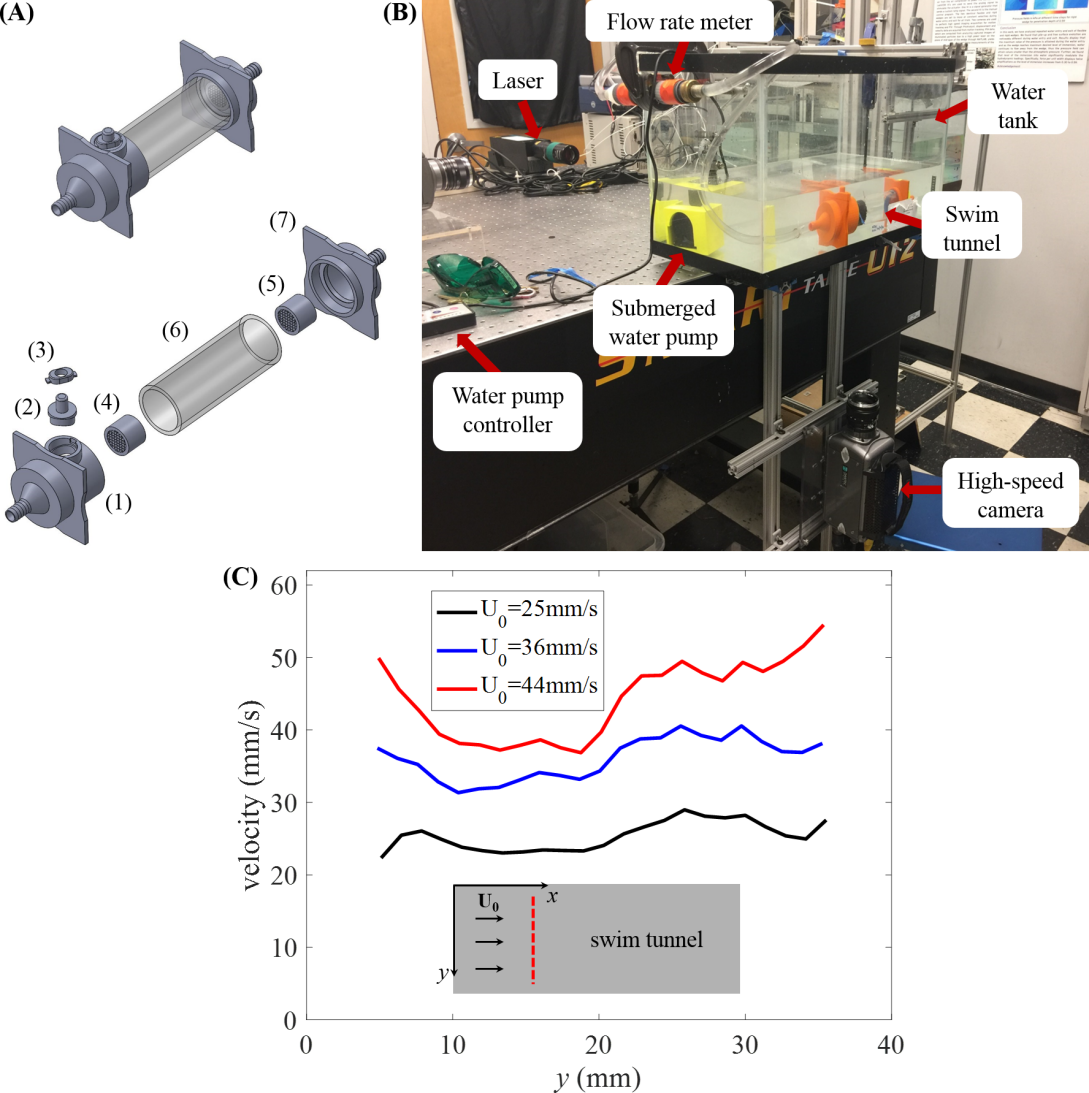
Experimental setup. (A) Assembled model of swim tunnel (top) and exploded view (bottom) of the parts, including a 3D printed funnel (1) to inject water flow with a small cylindrical hall frame serving as door frame, a 3D printed cap serving as a door (2), a 3D printed door locker (3), plastic collimators (4) and (5) placed on each side of the acrylic tube (6) in order to sustain laminar flow, and a second 3D printed funnel (7) to output the water flow. (B) Experimental setup for the study of zebrafish swimming against a water current. The camera is connected to an external computer to store the picture frames for subsequent analysis. (C) Flow velocity magnitude along the diameter (red dashed line) of the swim tunnel measured in the absence of the fish at three different flow rates.

A miniature swim tunnel (Fig. 1A) was designed and realized to generate laminar flow, similar to (Friedman, Liberzon & Kósa, 2015). The swim tunnel comprised a long acrylic tube measuring 15 cm long, with 5.72 cm outer diameter and 4.57 cm inner diameter, four 3D-printed components including two funnels (at the inlet and outlet), a door cap, and a locker, all of which were designed in SolidWorks (Dassault Systèmes SolidWorks Corp., Waltham, MA, USA) and fabricated using acrylonitrile butadiene styrene thermoplastic in a Dimension Elite three-dimensional printer (Stratasys Ltd., Eden Prairie, MN, USA).

The funnels measured 9.46 cm × 9.46 cm × 7.15 cm (length, width, and height, respectively) at the inlet of the acrylic tube and 9.46 cm × 9.46 cm × 11 cm at the outlet. Both funnels were lofted with an inside diameter of 1.16 cm, increasing to 4.57 cm at its outlet. The inlet funnel was connected to a 5 cm 3D-printed tube housing a miniature door of 3 cm diameter. The door was intended to facilitate the process of moving fish in and out of the swim tunnel and removing undesired water bubbles inside the tunnel. However, during the experimental sessions, we realized that it was more practical and less stressful for the fish to remove one of the two funnels placed at one end of the acrylic tube to move the fish in and out of the swim tunnel. The connection between the funnel and the acrylic tube was sealed using thread seal tape. The transparent acrylic tube served as the main compartment, in which the focal subjects were recorded during experiments. Two cylindrical plastic collimators were placed in the inlet and outlet funnel to sustain laminar flow.

The swim tunnel was connected to a submersible DC water pump (Jebao, Zhongshan City, China) through two pieces of PVC tubing of 1.27 cm diameter, and a flow meter (Gardena, Ulm, Germany) was used to calibrate the flow speed, as shown in Fig. 1B. All pieces except the flow meter were submerged in a tank measuring 61 cm × 31 cm × 31 cm. The tank was filled with water up to approximately 12 cm and the acrylic tube was completely submerged—in our experiments, no observable light distortions were caused by the acrylic tube.

### Apparatus

Experiments were conducted using a time-resolved planar PIV system comprising a 5 W adjustable continuous wave RayPower laser (Dantec Dynamics, Holtsville, NY, USA), illuminating neutrally buoyant Polyamid seeding Particles (PSP) of 50 µm (Dantec Dynamics, Holtsville, NY, USA), and a Phantom high-speed camera V.9.1 (Dantec Dynamics, Holtsville, NY, USA), mounted beneath the water tank to record fish motion from below. The seeding particles in our experiments had a Stokes number of Stk < 5 ×10^−5^, such that they could closely follow the fluid flow (Dring, 1982; Melling, 1997). The high-speed camera was set at a resolution of 1,200 × 496 pixels, corresponding to a pixel size of 80 µm, to record images of fish swimming at 160 frames per second. The resolution and acquisition rate were sufficiently high to resolve detailed flow structures and fish tail beating both spatially and temporally. Specifically, from the literature, we expected adult zebrafish to have a tail beating frequency on the order of 20 Hz (Fontaine et al., 2008), tail beating amplitude during bursting on the order of 10 mm (Gilbert, Zerulla & Tierney, 2014), and vortex core radius on the order of 5 mm (Müller, Stamhuis & Videler, 2000).

The sides of the tank, except for the ones facing the camera and the laser, were covered with a black curtain to minimize outside visual influence on the subjects. The horizontal plane in the center of the tank was illuminated by a laser light sheet with a power of 1 W (light sheet approximately 0.5 mm thick, wavelength *λ* = 532 nm), which was sufficient for illuminating the particles while reducing possible disturbance to the fish.

### Experimental procedure

Experiments were performed from August to September 2016 between 10AM to 1PM and 3PM to 6PM. Water temperature was controlled at 27 °C using a water conditioner (Stress Coat+; Fishcare North America, Chalfont, PA, USA) and an auto-digital water heater (Aquatop, Brea, CA, USA). For each experiment, a fish was transferred from the housing tank to a beaker using a hand net, where it stayed for about 5 min before being transferred in the swim tunnel. The fish was left swimming for a habituation period of 5 min at a flow speed of 26 mm/s.

After the habituation period, the laser was turned on to illuminate the mid horizontal plane of the water tunnel and the camera was set to record simultaneously. The recording process lasted about 20 s before the laser and camera were turned off. After the first recording, the water flow was increased to a speed level of 39 mm/s, and 5 min of habituation time was given to the fish prior to the next recording of 20 s. Identical steps were repeated for a third speed level with the water flow set at 52 mm/s. After the third experimental condition, the fish was moved from the swim tunnel to the beaker and then placed in a separate housing tank. The same procedure described above was repeated for a total of 12 zebrafish.

The increasing speed of the water and the large habituation period between trials were chosen to mitigate fatigue in experimental subjects. For each speed level, whenever the fish was found at the laser plane, the trial was marked as success, otherwise it was marked as failure. In our study, we only analyzed instances where the whole fish body appeared in the field of view, indicating that the fish was swimming in the laser plane. To mitigate the influence of spatial variations in the water velocity close to the wall, we discarded instances in which fish swam in close proximity to the wall. Table 1 provides a summary of the experiments and their success rate for all the 36 trials.

**Table 1:** Summary of the experiments and the available data for analysis of zebrafish swimming behaviors.

	26 mm/s	39 mm/s	52 mm/s	Total
Number of trials	12	12	12	36
Success rate	11/12	6/12	9/12	26/36
Number of burst instances	10	6	8	24
Number of cruise instances	7	5	9	21

### Flow visualization

The raw video frames were processed using PIVLab (Thielicke & Stamhuis, 2014), an open source digital PIV post-processing package programed in MATLAB R2015a (Mathwork, Natick, MA, USA) to obtain the velocity data. The PIV analysis consisted of fast Fourier transform with decreasing interrogation window size of 64 × 64, 32 × 32, and 16 × 16 pixels (Scarano & Riethmuller, 1999), an interrogation window overlap of 50%, and 2 × 3 Gaussian subpixel interpolation (Thielicke & Stamhuis, 2014). Calibration was performed using a checkerboard with 8 × 8 mm^2^ sized squares. An in-house image processing program was written in MATLAB to extract fish body kinematics from the videos and vorticity information from the velocity data. Before our experiments, the flow field in the swim tunnel without the fish was measured.

From these measurements, we found substantial variations in the flow speed near the wall of the water tunnel, which prompted the elimination of instances of swimming close to the wall that we have mentioned above. The velocity profiles along the diameter away from the wall of the swim tunnel at different flow rates are displayed in Fig. 1C, which demonstrates reasonably uniform flow velocity across the cross-section of the tunnel.

### Analysis

**Figure 2:**
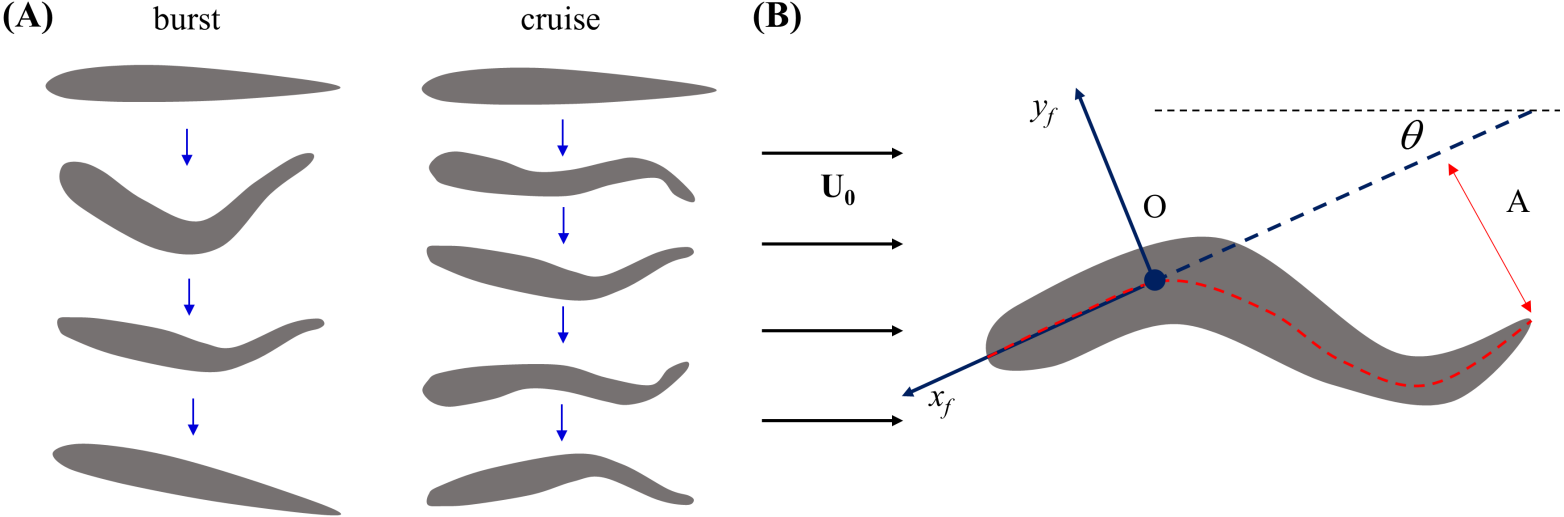
Illustration of zebrafish locomotion. (A) Schematics of fish body shape undergoing burst and cruise. (B) Schematics of a swimming fish in the water tunnel. The central line of the body is represented by the red dashed line. A fixed point (O) on the fish body is chosen as 1/3 of the body length from the head. The heading direction (*x*_*f*_) of the fish is determined by a line connecting point O and the tip of the head. A coordinate system attached to the fish body with origin at O is indicated by the blue arrows. The fish tail beating amplitude (*A*) is tracked in the body frame.

To analyze zebrafish swimming, we considered two main locomotory patterns (Fig. 2A): (i) burst, defined as an isolated body undulation, followed by a coasting phase, in which the body of the fish would not bend, and (ii) cruise, defined as continuous tail beating to propel the fish forward or to sustain a relatively fixed position against the current. Locomotory patterns that could not be robustly classified as burst or cruise were not considered in the analysis. For example, coasting instances, during which fish ceases any tail beat movement, were not analyzed.

#### Fish locomotion

The effect of flow speed *U* (mm/s) on fish swimming kinematics was evaluated using the tail beat amplitude *A* (mm), tail beat frequency *f* (1/s), and change in the heading angle Δ*θ* (rad). These variables were all extracted from the fish body on consecutive frames using an in-house custom developed program using MATLAB image processing toolbox.

Briefly, the fish body shape was fitted by a curve through its body mid-line. Then, fish position in the tank inertial frame was tracked by selecting a single fixed point (O) on the fish body over time. This point was chosen such that the length of the mid-line from O to the tip of the head was one third of the whole body length (*L*), which in the literature has been found to be approximately rigid during swimming (Fontaine et al., 2008). Similar to the work of Müller, Stamhuis & Videler (2000), we then defined a coordinate system in the image plane attached to the fish body with origin O, comprising an *x*_*f*_-axis from O toward the tip of the head and a *y*_*f*_-axis orthogonal to the *x*_*f*_-axis, pointing out from the right lateral side (Fig. 2B). Fish heading direction with respect to the tank inertial frame was computed as the angle between the tunnel length and the fish *x*_*f*_-axis. Tail beat amplitude *A* was computed as half of the maximum tail tip displacement during each tail beating cycle in the fish body frame (Fig. 2B). In case of a burst, *A* was calculated from a single tail beating cycle, while for a cruise, we considered the average value of *A* over all the observed tail beat cycles. Tail beat frequency *f* was computed as the inverse of the duration of a complete tail beat cycle for a burst, while in case of a cruise *f* represents the averaged value over all tail beat cycles.

The velocity of the fish relative to the flow is defined as **V** = **U**_**0**_**-u**, where **U**_**0**_ is the upstream flow velocity and **u** is the fish velocity with respect to the tank inertial frame, computed by taking the first time derivative of the time trace of the trajectory data of point O. Here and in what follows, we use bold letters to denote vectors and italic fonts for their magnitude.

Fish swimming speed relative to the flow (|**V**| = *V*), tail beat amplitude, tail beat frequency, and heading angle change were computed for all successful trials in the three speed conditions (26 mm/s, 39 mm/s, 52 mm/s) and for the two selected locomotory patterns, that is, burst and cruise. Within a fluid dynamics framework to study zebrafish swimming, we also aggregated the previous indicators in two key nondimensional numbers (Gazzola, Argentina & Mahadevan, 2014). To quantify the interplay between viscous and inertial phenomena, we introduce the Reynolds number, defined as Re = *ρVL*∕*μ*, where *ρ* is the fluid density and μ is the fluid viscosity. Toward a single measure to describe the combined effect of tail beating and body swimming speed for both cruising and bursting instances, we define the Strouhal number as St = *fA*∕*V*.

To test the effect of the flow speed and the locomotory patterns on the measured indicators, two-way analyses of variance (two-ways ANOVA) were performed with locomotory patterns (burst and cruise) as between factor and flow speed (26, 39, and 52 mm/s) as within factor. In case where the flow speed was observed to significantly affect the value of an indicator, Tukey’s Honest Significant Difference (HSD) post-hoc test was conducted to differentiate between speed levels. The effects of flow speed and locomotory patterns on the aggregated nondimensional numbers, Re and St, were also assessed using two-way ANOVA with fish locomotory patterns as between factor and flow speed as within factor. Tuckey’s HSD post-hoc test was conducted to differentiate between speed levels. Unless otherwise specified, all the statistics are evaluated at *p* < 0.05 significance level.

A Generalized Liner Regression fit (GLM) was also performed between Re and St to explore the dependence of St on Re. The linear regression slope of the regressions obtained by differentiating between locomotory patterns was compared to further assess the effect of the swimming style using the methodology described by Clogg, Petkova & Haritou (1995).

#### Flow physics

Salient information on the flow physics were extracted from the velocity and the vorticity fields obtained from PIV. The circulation associated with vortex shedding was estimated along a closed curve (*C*) as defined in the literature (Saffman, 1992; Lim & Nickels, 1995)

(1) \begin{eqnarray*}\Gamma ={\oint }_{C}\mathbf{U}\cdot d\mathbi{l},\end{eqnarray*}

where *d****l*** is an infinitesimal segment of curve *C* and **U** is the velocity field. Since our data were recorded in two-dimensions, the above formula was converted to a two-dimensional integral over the area (Ω) enclosed by *C*, which could be approximated on the discretized PIV mesh as

(2) \begin{eqnarray*}\Gamma ={\iint }_{\Omega }{\omega }_{z}dS\approx \sum _{i}{\omega }_{zi}\Delta S,\end{eqnarray*}

where *ω*_*z*_ (1/s) is the out-of-plane component of the vorticity field, *ω*_*zi*_ is the *ω*_*z*_ value at grid point *i* of the PIV mesh, and Δ*S* is the area of each discretized mesh element. GLM was used to explore the dependence of the circulation on the Strouhal number and a similar analysis was conducted on change in the heading angle Δ*θ* to illustrate how vorticity shedding would relate with change in swimming direction.

The pressure field was reconstructed from the fluid velocity field around the fish using a method similar to that employed by Panciroli & Porfiri (2013). Briefly, the flow physics in the water tunnel was described by incompressible Navier–Stokes equations relating the fluid velocity to the pressure gradient. By integrating the velocity field, the pressure gradient distribution was indirectly computed. The fluid pressure was then extracted by integration. In this study, we first integrated the velocity field along the fluid boundary to obtain the pressure on the boundary. The pressure in the fluid domain was then reconstructed by integration from the boundary toward the bulk along set directions. To minimize accumulation errors, we followed the approach in Dabiri et al. (2014), such that the velocity field was integrated along eight different directions (two horizontal directions, two vertical directions, and four diagonal directions), and the eight resulting pressure fields were averaged into a single field.

## Results

### Influence of flow speed on fish locomotion

**Figure 3:**
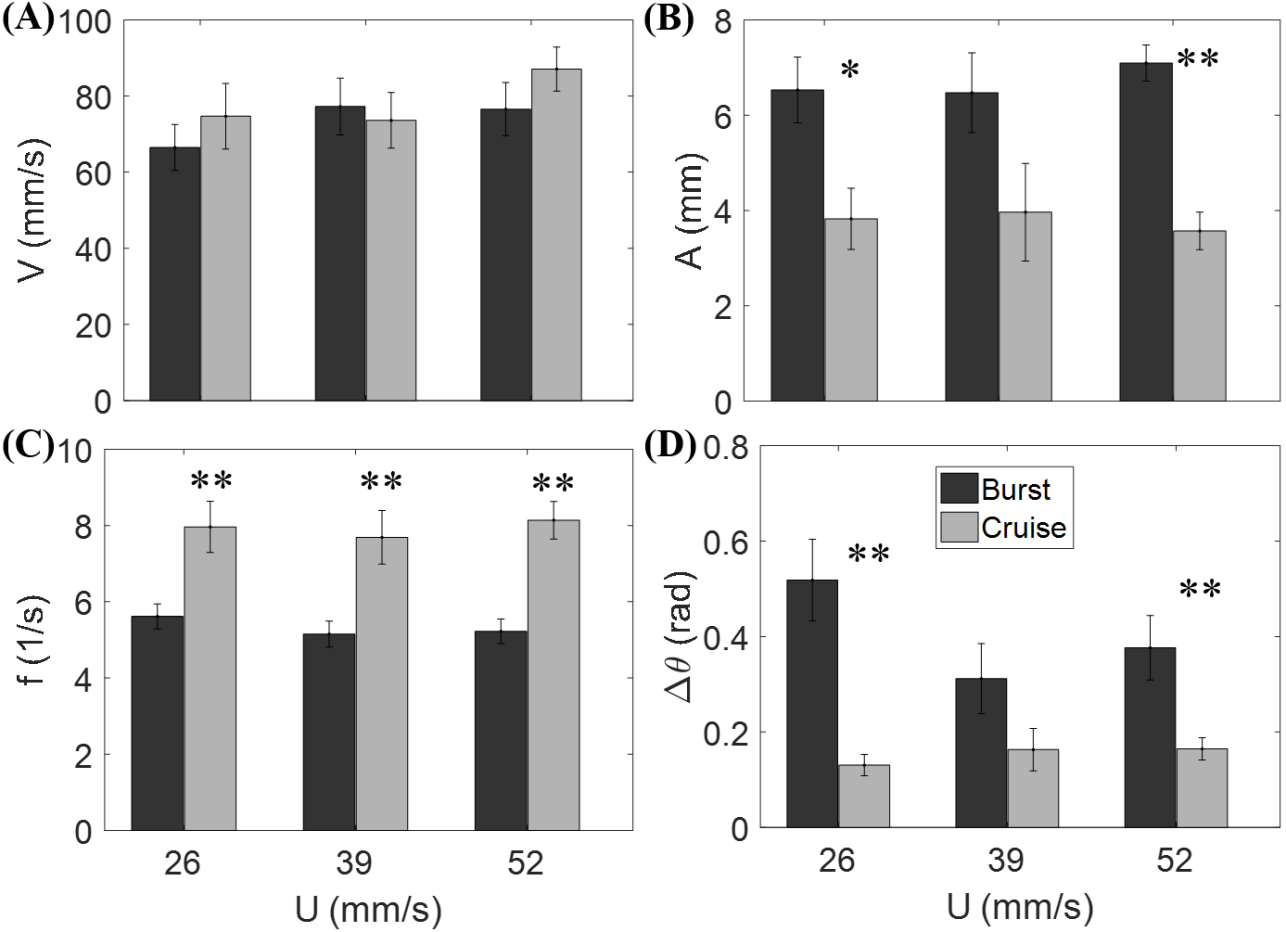
Measured locomotory indicators of zebrafish swimming. Comparison of (A) fish swimming velocity, (B) tail beat amplitude, (C) tail beat frequency, and (D) change in heading angle for both burst and cruise at three flow speeds (*U* = 26, 39, 52 mm/s). Label ^∗^ indicates significant difference between burst and cruise at *p* < 0.05, and ^∗∗^ is for significance at *p* < 0.01. Error bars represent ± standard errors.

Figure 3 shows that the flow speed does not significantly affect tail beat amplitude (*F*_2,44_ = 0.04, *p* = 0.964), tail beat frequency (*F*_2,44_ = 0.30, *p* = 0.740), relative velocity (*F*_2,44_ = 1.53, *p* = 0.228), and change of heading direction (*F*_2,44_ = 1.27, *p* = 0.293). However, fish locomotory pattern was observed to significantly influence tail beat amplitude (*F*_1,44_ = 31.33, *p* < 0.001), tail beat frequency (*F*_1,44_ = 45.09, *p* < 0.001), and change of heading angle (*F*_1,44_ = 25.17, *p* < 0.001). In most cruising instances, fish were observed to swim upstream, such that the fish were able to swim faster than the flow.

### Nondimensional numbers of fish locomotion

**Figure 4:**
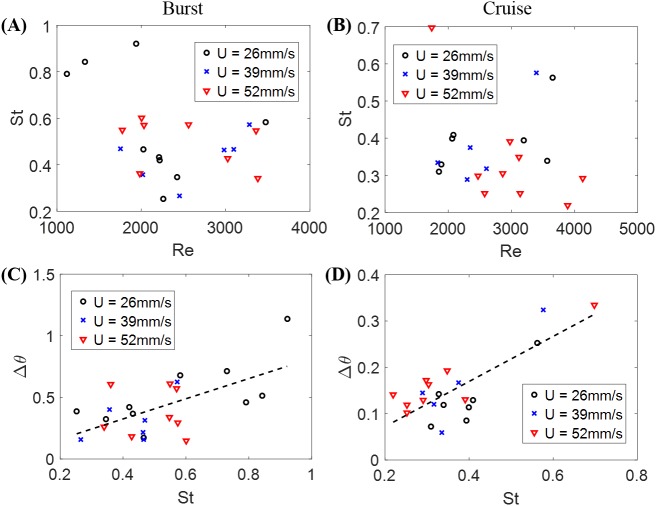
Nondimensional numbers associated with zebrafish locomotion. Correlation between the Strouhal number St and the Reynold number Re for (A) burst and (B) cruise and correlation between fish change in heading angle (Δ*θ*) and St for (C) burst and (D) cruise. Dashed black lines in the figure indicate estimated linear regression fit of Δ*θ* = 0.819 St − 0.002, with *p* = 0.002. The dashed black line in (D) is a linear regression fit of Δ*θ* = 0.487 St − 0.025, with *p* < 0.001.

The Strouhal number St was 0.366 ± 0.026 when cruising, compared to St = 0.515 ± 0.035 during bursting. Fish swimming was further explored by studying the relation between St and Re in Figs. 4A and 4B for burst and cruise. A generalized linear regression fit for the data displayed in Figs. 4A and 4B does not suggest a dependence between St and Re (*F*_1,22_ = 1.34, *p* = 0.259 for burst; *F*_1,19_ = 0.32, *p* = 0.578 for cruise).

The relation between St and Δ*θ* for burst and cruise is shown in Figs. 4C and 4D. In the figure, the values of Δ*θ* are observed to significantly increase as St increases for burst (Δ*θ* = 0.819 St − 0.002, with *F*_1,22_ = 12.80, *p* = 0.002, Dispersion = 0.0362) and cruising (Δ*θ* = 0.487 St − 0.025 with *F*_1,19_ = 31.20, *p* < 0.001, Dispersion = 0.0021). Introducing a dummy variable in the generalized linear regression model to test the mixed effects of both behaviors, we determined that the two linear regression models have significantly different slopes (*F*_3,41_ = 21.70, *p* < 0.001, Dispersion = 0.0204).

### Flow physics around the fish

**Figure 5:**
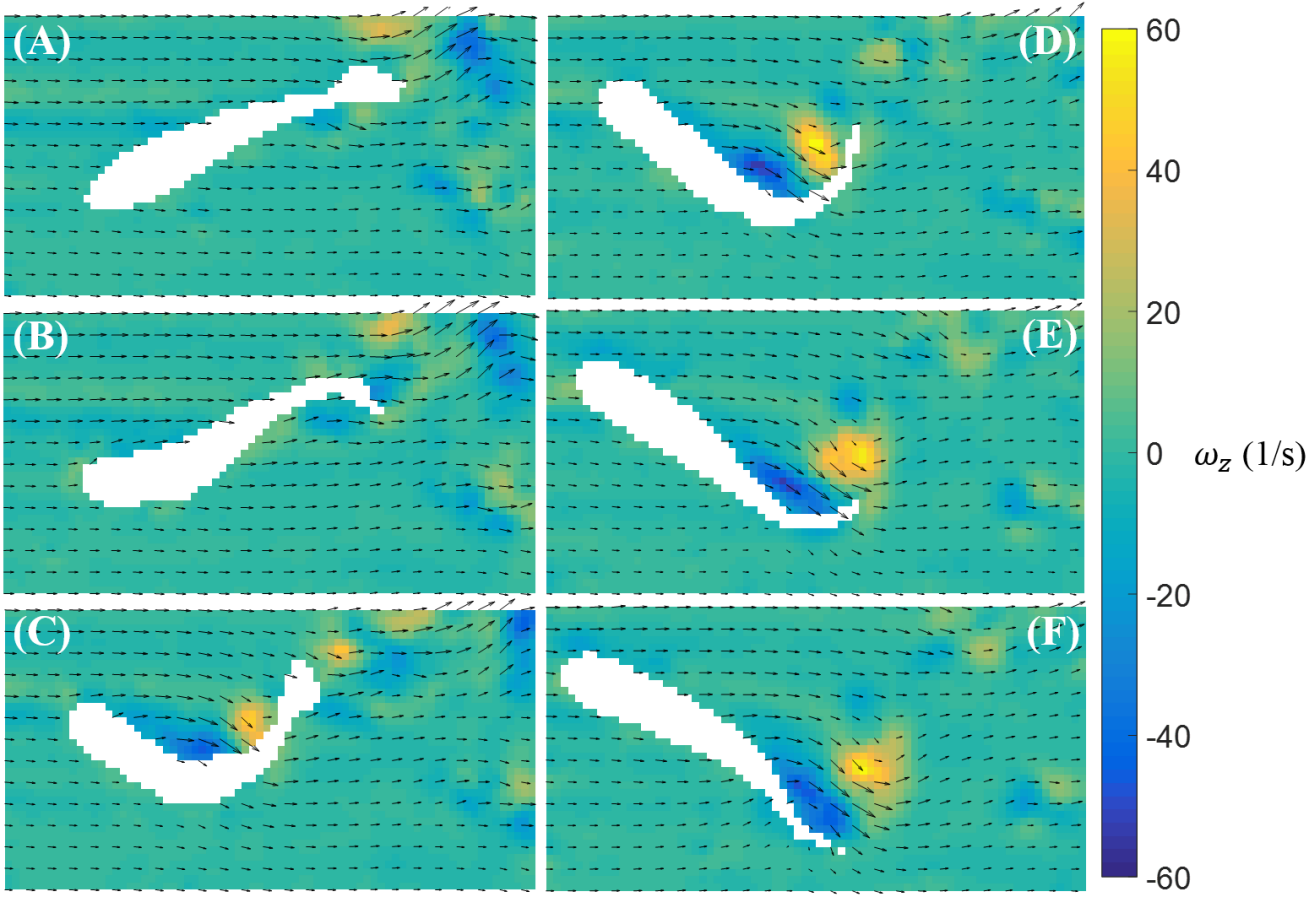
Velocity and vorticity fields around a bursting zebrafish. Time sequence (A–F) of the velocity (arrow) and vorticity (color) fields in the vicinity of the fish body during a burst against a laminar flow with speed *U* = 39 mm/s. The time interval between consecutive snapshots is 1/32 s. For visualization purposes, one velocity vector is plotted every three rows and columns.

In Fig. 5, we illustrate a typical vorticity field observed over the duration of a burst. Note that for visualization purposes, one velocity vector is plotted every three rows and columns for all velocity fields presented in this paper. To initiate a burst, the fish bends its body with a large curvature. This body motion creates a pair of vortices with opposite signs near the middle of the body (Figs. 5A–5C). The fish tail then beats in the opposite direction and releases a pair of vortices downstream (Figs. 5D–5F).

**Figure 6:**
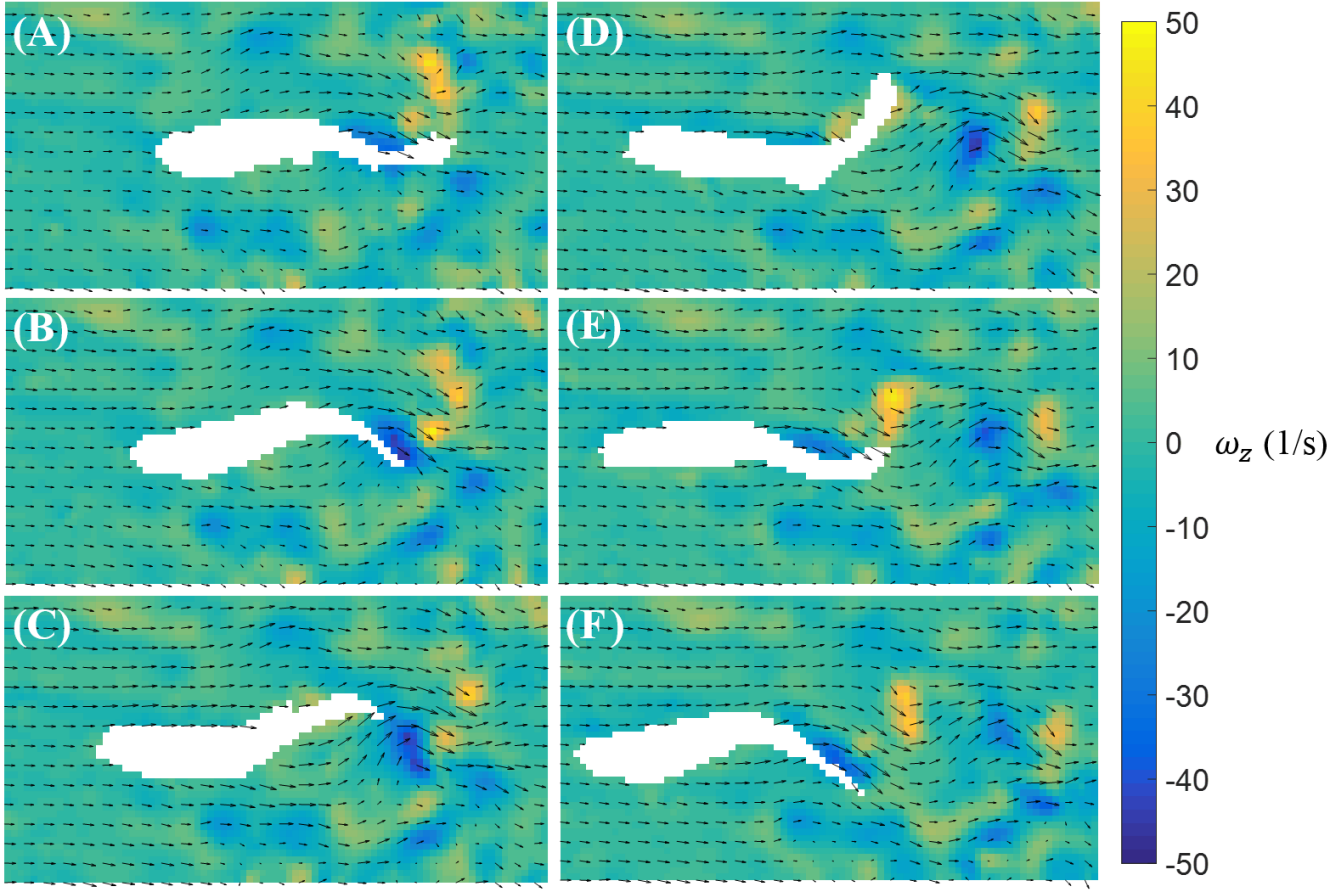
Velocity and vorticity fields around a cruising zebrafish. Time sequence (A–F) of the velocity (arrow) and vorticity (color) fields in the vicinity of the fish body during a cruise against a flow with speed *U* = 26 mm/s. The time interval between consecutive snapshots is 1/32 s. For visualization purposes, one velocity vector is plotted every three rows and columns.

In contrast to burst, the magnitude of the body bending and tail beating of a cruising fish is smaller, as illustrated in Fig. 6. One cycle of tail beat during cruising is exhibited in Fig. 6, which shows that the fish heading direction is relatively steady over time. Vortices with alternating signs are shed downstream of the fish over the course of a cruise instance.

**Figure 7:**
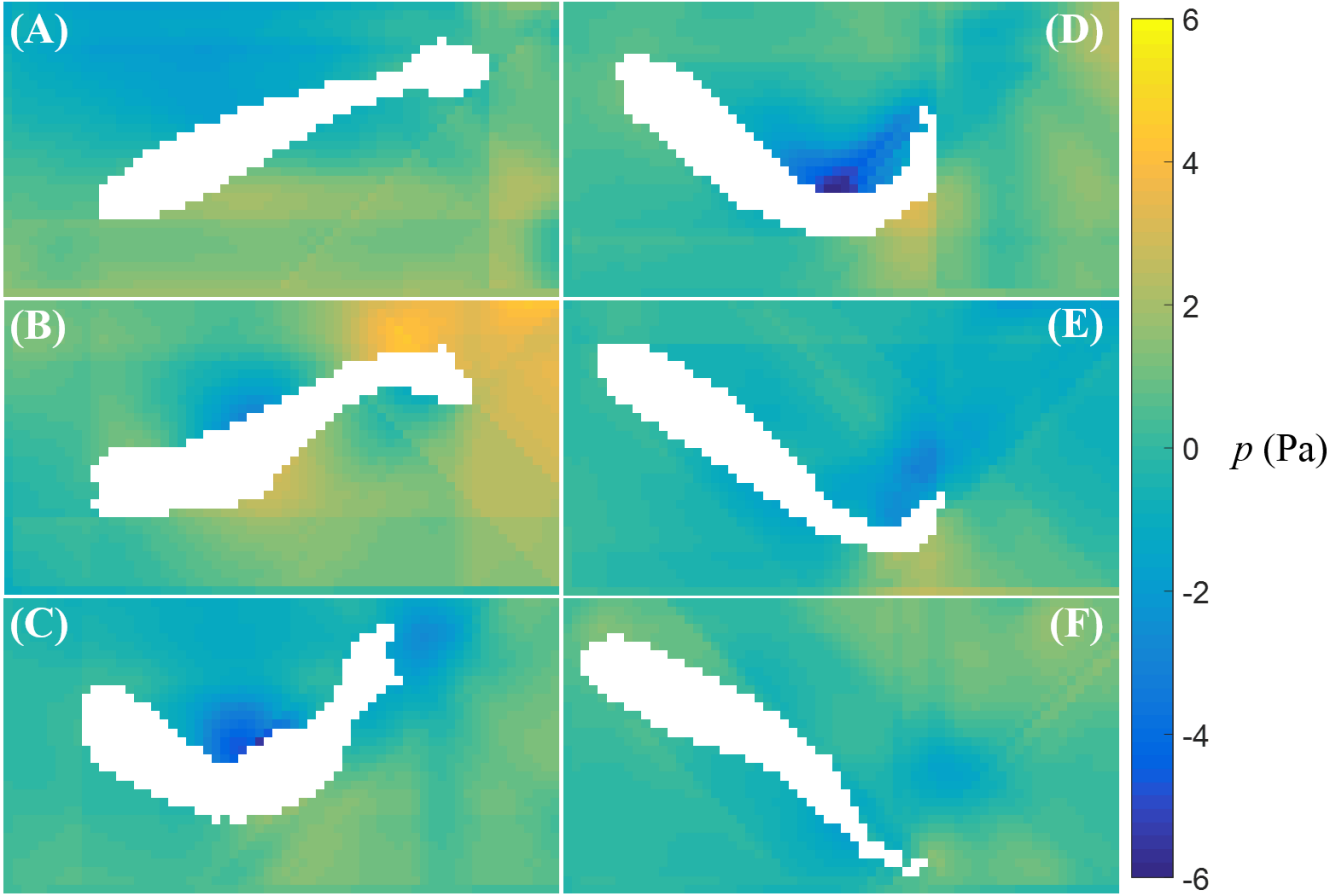
Fluid pressure around a bursting zebrafish. Fluid pressure fields (A–F) correspond to the velocity fields shown in Figs. 5A–5F.

**Figure 8:**
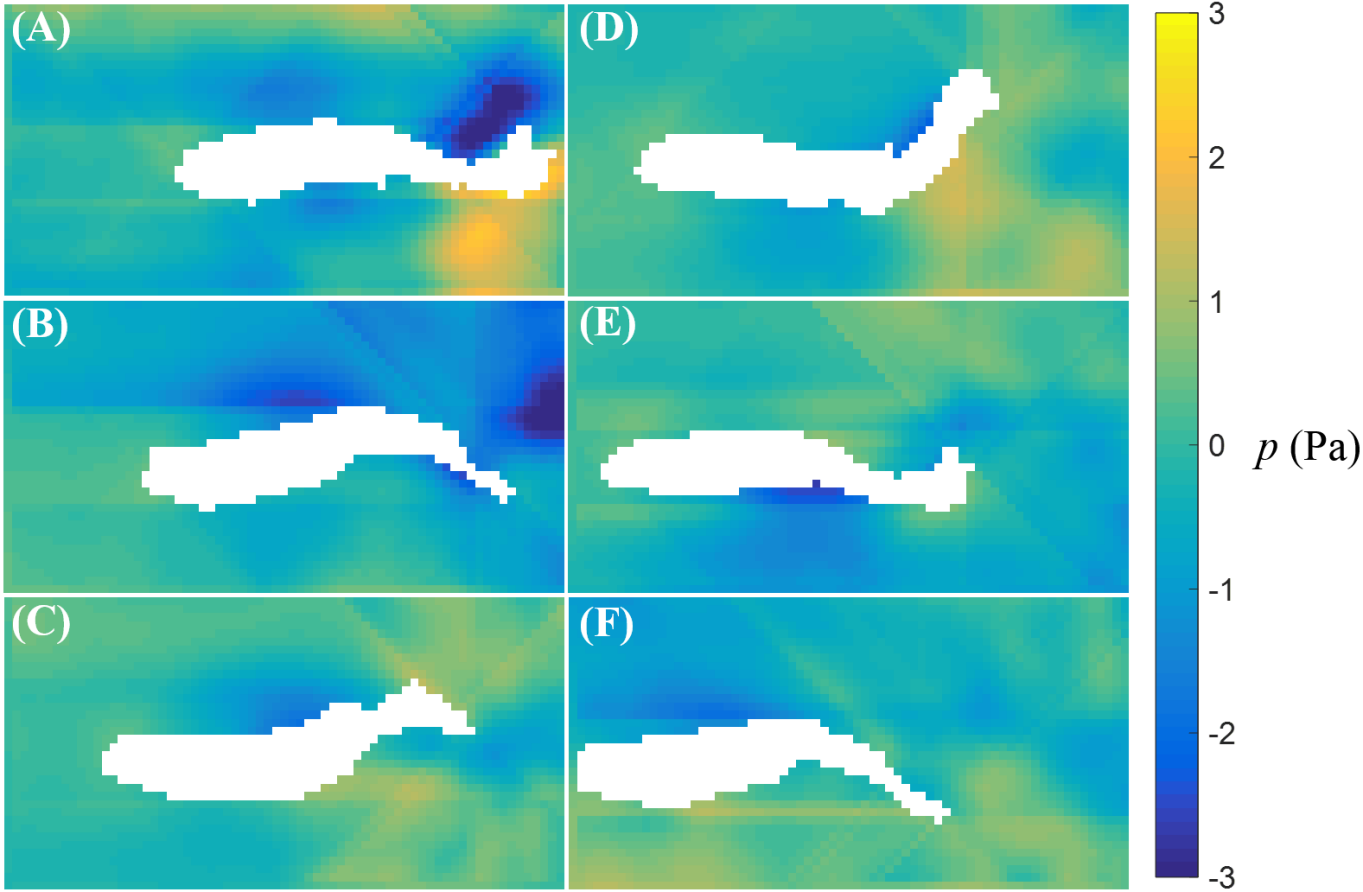
Fluid pressure around a cruising zebrafish. Fluid pressure fields (A–F) correspond to the velocity fields shown in Figs. 6A–6F.

The fluid pressure fields around a bursting and cruising fish are displayed in Figs. 7 and 8, respectively. During a burst, as the tail pushes the fluid sideways away from the body, a high pressure region is created ahead of the tail, and a low pressure region is created in the back, as shown in Figs. 7C–7E. This results into a net force that has a positive component along the fish heading direction, which leads to a propulsion on the fish body. A similar phenomenon can be observed during cruising (Figs. 8C–8E). The pressure magnitude around the tail during cruising is generally smaller than bursting.

**Figure 9:**
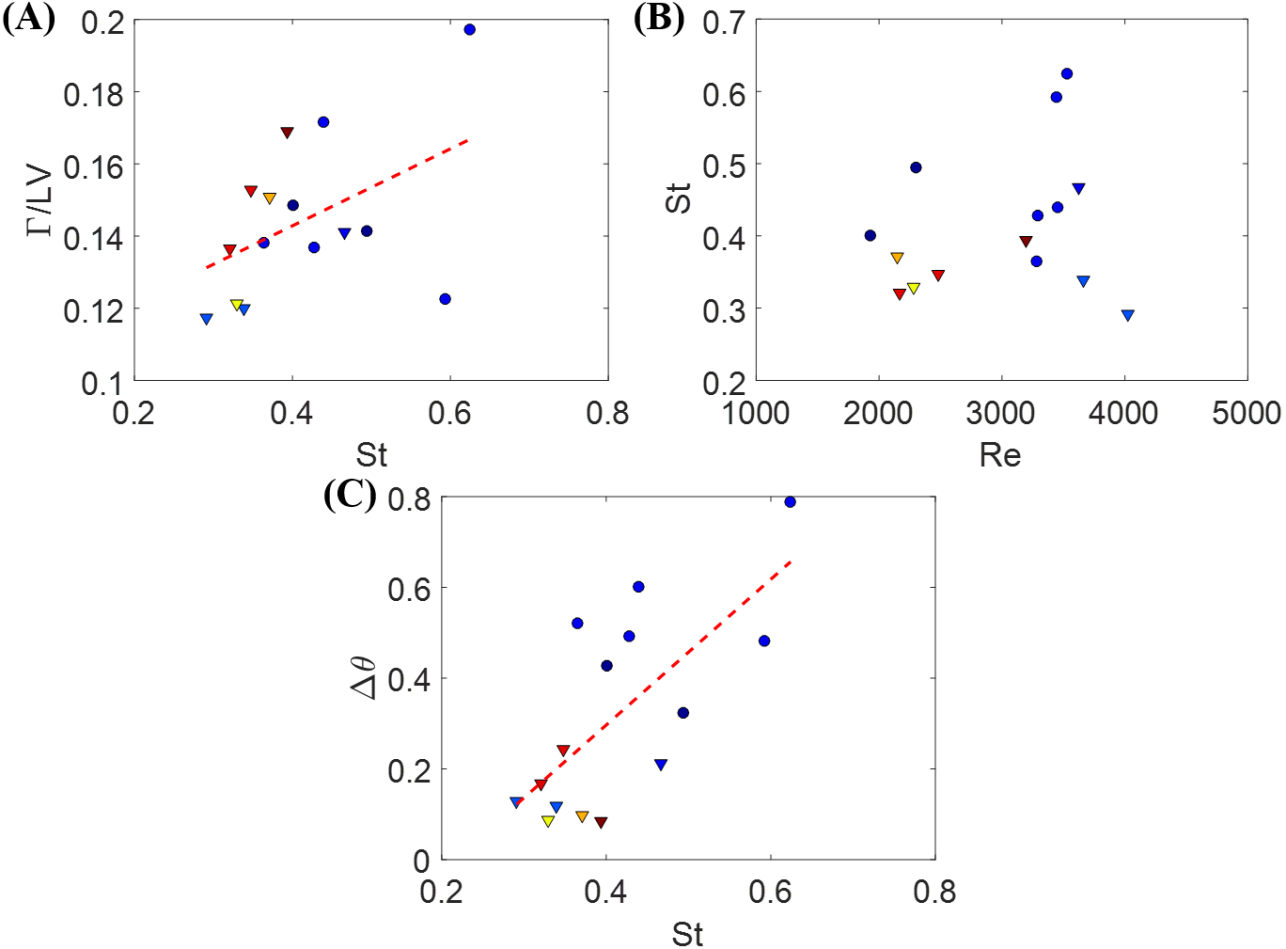
Measurements from individual zebrafish burst and cruise movements. (A) Normalized circulation as a function of the relative Strouhal number. Dashed line is a linear fit to the data points, Γ∕*LV* = 0.11St + 0.10, with *p* = 0.078. Triangles represent cruise movements while circles represent burst movements. Each data point is obtained based on one movement of burst or cruise. Data from different fish are plotted in different colors. (B) St as a function of Re for the same movement presented in (A). (C) Δ*θ* as a function of St for the same instances presented in (A). Dashed line is a linear fit to the data points, Δ*θ* = 1.61St − 0.346, with *p* = 0.003.

The circulation of the vortices shed by the fish for different St values is presented; each data point represents a single instance of burst or cruise (Fig. 9). A generalized linear regression between the circulation Γ∕(*LV*) and St does not permit to validate the hypothesis of a linear relation (*F*_1,13_ = 3.68, *p* = 0.077). In addition, combining the available data (when the fish was in the laser plane) for both burst and cruise, we found no linear dependence between St and Re using a generalized linear regression (*F*_1,13_ = 0.71, *p* = 0.416). Consistent with the results shown in Figs. 4A and 4B, using a linear regression fit, we observed that Δ*θ* is positively correlated to St (Δ*θ* = 1.61St − 0.346, with *F*_1,13_ = 12.9, *p* = 0.003).

## Discussion

In this work, we studied the locomotion and hydrodynamics of zebrafish swimming against a water current. Locomotory patterns typically observed in placid water, that is, burst-and-coast movements, were also recorded in the water channel. Driven by the continuous water flow, zebrafish were also found to cruise frequently. Zebrafish locomotion was evaluated by several indicators, including tail tip kinematics, relative velocity, and change of heading direction. Statistical analysis revealed that zebrafish locomotory patterns were not significantly altered by the flow speed in the water tunnel. This may be explained by the observation that zebrafish could swim at a higher speed than the flow at all tested speeds in our experiments. Fish may thus overcome drag induced by the incoming flow without the need of adjusting locomotory pattern.

However, locomotory patterns, burst and cruise, were observed to significantly influence the swimming indicators considered in the study. In particular, bursting fish experienced larger tail beat amplitude, lower tail beat frequency, and larger heading direction change compared to cruising fish. The differences between burst and cruise may originate from the muscles used to perform these two types of motions. Based on the measurement of muscle length and stimulation patterns by electromyography (EMG), past experiments have associated cruise motions with red (slow) muscle fibers, and burst motions with white (fast) muscle fibers (Rome et al., 1988; Rome, Swank & Corda, 1993; Wakeling, 2001). The location, orientation, and shortening speed of white and red muscles during burst and cruise motions might help explaining the difference in the locomotory patterns (Johnston, Davison & Goldspink, 1977; Rome et al., 1988; Rome, Swank & Corda, 1993; Wakeling, 2001; Müller & Van Leeuwen, 2006) and provide insights in the propulsive efficiency of the two swimming patterns (Rome et al., 1988). Direct EMG measurement of zebrafish muscle activities would be helpful to clarify the relation between muscle actuation and burst and cruise motions in a water current.

Our computation of St based on the fish tail beat amplitude and frequency and the relative fish swimming speed indicated that St was independent of Re. This is consistent with the scaling law for aquatic locomotion proposed by Gazzola, Argentina & Mahadevan (2014), where St values of swimmers at Re spanning seven orders of magnitude were acquired and the value St ≈ 0.3 was found to be independent of Re for Re > 10^4^, and a weak dependence, St ∼ Re^−1∕4^, was discovered for Re < 10^4^. We found that when cruising against a current, zebrafish swam in the range of 0.2 < St < 0.4, which is in agreement with values reported in the literature for other animals (Triantafyllou, Triantafyllou & Yue, 2000; Taylor, Nudds & Thomas, 2003; Gazzola, Argentina & Mahadevan, 2014). Based on the technical literature (Triantafyllou, Triantafyllou & Grosenbaugh, 1993; Triantafyllou, Triantafyllou & Yue, 2000), we may hypothesize that this provides a high propulsive efficiency to zebrafish. This claim rests on experiments on oscillating foils which have demonstrated that the propulsive efficiency, defined as the ratio of the hydrodynamic thrust power to the input power (Triantafyllou, Triantafyllou & Grosenbaugh, 1993; Triantafyllou, Triantafyllou & Yue, 2000), peaks at St ≈ 0.3.

In our experiments, we were able to resolve full tail beating cycles of bursts and cruises, as well as the flow physics with sufficient resolution. Similar to the vorticity field shown around a bursting zebrafish in placid water where a pair of vortices was produced by sharp body bending (Müller, Stamhuis & Videler, 2000), we found that when bursting in a water current, a pair of vortices was generated near the middle of the body as it bent. The vortices detached from the tail and travelled along the flow as the tail beat. In contrast, the steady tail undulations during cruising caused the continuous generation of vortices in the wake of the body. During fish tail undulation, vortices with alternating signs were created and advected on both sides of the tail trajectory. The visualized vortical structures can be categorized as either a chain of connected vortex rings or a double-row of isolated vortex rings intersecting the laser plane (Blickhan et al., 1992; Müller et al., 2001; Müller & Van Leeuwen, 2006). A vortex chain would appear on a two-dimensional plane as series of vortices with alternating signs distributed on both sides of the tail tip moving trajectory (Müller et al., 2001), which matches our observations. A double-row of vortex rings, on the other hand, should produce two rows of double vortices, which is not observed in our results. We may assume that when cruising against the water current, zebrafish form vortex chains by continuous tail beating. This observation is different from the vortical structures observed in zebrafish larvae cruising in a more viscous fluid, which produced two rows of double vortices (Müller, Van den Boogaart & Van Leeuwen, 2008). Future work should seek to extend our study to three dimensions to further elucidate the structure of the wake generated by zebrafish. These experiments might help the classification of the vortical structures and improve on the data collection.

The flow visualization presented in this study revealed detailed flow features induced by zebrafish swimming against a water current. Similar wake structures have been observed in some other fish species during burst and cruise movements. PIV studies on bursting giant danio (*Danio aequipinnatus*) have revealed two pairs of vortices shed during a burst: a weak pair of vortices shed when the fish forms a C-shape, similar to the fish body shape shown in Fig. 5C, and a strong pair of vortices shed toward the end of the burst (Epps & Techet, 2007). From our PIV results, it is not clear whether a weak pair of vortices is formed during the burst of zebrafish, due to the level of noise in the vorticity data; however, a strong pair of vortices has been found to initiate near the center of the body and shed by the tail. The values of the nondimensional circulation (Γ∕(*LV*)) of the vortices generated by zebrafish are on the same order of magnitude as those induced by cruising giant danio. In addition, the vortical patterns generated by cruising zebrafish against a current are similar to the wake structure of a steady swimming mullet (*Chelon labrosus*), which also features a series of vortices with alternating signs (Müller et al., 1997). In contrast, two rows of double vortices are observed in the wake of eel (*Anguilla anguilla*) during steady swimming, which might be employed to achieve high maneuverability (Müller et al., 2001).

In our work, the pressure field around the swimming fish was reconstructed by direct integration of the velocity field. The pressure distribution around the fish tail showed a net propulsive force on the fish body during burst and cruise motions. The magnitude of the pressure on the fish was higher during burst than cruise. In the literature, fluid pressure around swimming zebrafish has been quantified only with computational fluid dynamics simulations, where burst and cruise movements were simulated and the thrust was computed (Katumata, Müller & Liu, 2009). These simulations have shown that a higher propulsive force is produced by burst than cruise, which is consistent with our findings. Pressure reconstruction techniques have also been employed to quantify the pressure field around swimming lampreys (*Petromyzon marinus*) and jellyfish (*Aurelia aurita*) (Dabiri et al., 2014; Gemmell et al., 2015), thereby clarifying the role of suction in thrust production during swimming. Compared to our results, smoother pressure distributions have been reported in these studies, which may be due to a faster temporal PIV resolution and a larger particle image pixel size. A faster temporal resolution may lead to a more accurate estimation of the fluid acceleration effect, and large image pixels might have helped smoothing the velocity field. Different from experimental models in robotics (Zhang et al., 2016), where a swimming robot can be instrumented with pressure sensors, our understanding of live fish locomotion depends on non-invasive measurements and this work puts forward a first characterization of the pressure field induced by zebrafish swimming.

The circulation associated with the vortices shed during both bursting and cruising movements showed a weak dependence on St: tail beats with higher St values in most cases resulted in vortices with higher circulations. Using the linear relation between circulation and impulse derived in Saffman (1992) and Lim & Nickels (1995), we might infer that a vortex with a higher circulation produces a higher impulse on the fish body. Since most bursts were associated with high St values, we may propose that compared to cruise, fish gained higher impulse through burst during each tail beat cycle. This observation is consistent with the functionalities of these two types of movements (Wakeling, 2001; Müller & Van Leeuwen, 2006). The high impulse gained during a burst was utilized by the fish to change its heading direction and body location in a swift manner, while the impulse generated by a cruise was used primarily to overcome the drag from the water current and maintain steady swimming.

A number of limitations exist in the present study, which will be addressed in future works. First, in our experiments, an increasing speed for the water flow was chosen for all fish. While doing so was intended to minimize fatigue in experimental subjects, we acknowledge that randomizing the order of the three flow speeds could help reduce bias in the experimental conditions. Second, the linear acceleration of zebrafish during cruise and burst was not taken into consideration in our analysis due to the high level of noise introduced by differentiating twice the fish position. Acceleration during swimming may indicate a higher propulsion generated by tail beating compared to steady swimming (Domenici & Blake, 1997). Third, the reconstructed flow pressure around the fish body contains error pertaining to line integrations around the fish body. This error might be reduced by implementing a Poisson formulation for the pressure field (Van Oudheusden, 2013; McClure & Yarusevych, 2017), but this would require an improved knowledge of pressure boundary conditions. Fourth, during our two-dimensional velocity measurement, a vortex is often seen breaking up into two vortices, which would eventually disappear in the downstream of the flow. This might result from misalignment between the fish and the laser plane, and could perhaps be resolved through volumetric measurements. Finally, future work might seek to complement this study with a control condition with fish swimming in still water in order to differentiate the effect of the flow on zebrafish locomotory pattern.

In this study, we have attempted to elucidate how a water current may affect the locomotion of adult zebrafish. Water currents are prevalent in zebrafish ecological niche, characterized by secondary and tertiary channels connected to main rivers where the flow speed can reach several body lengths per second (Arunachalam et al., 2013). Our work demonstrated the coexistence of different locomotory patterns that are employed by zebrafish in such a dynamic environment. Through the measurement of St, we have shown that cruising against a uniform current follows the well-known power law observed across different swimming species (Gazzola, Argentina & Mahadevan, 2014). The flow measurement in our experiments also revealed different vortical structures and hydrodynamic pressure distributions induced by burst and cruise, which may be explained by the different functionalities of these locomotion patterns. This study may help to elucidate how zebrafish utilize different locomotory patterns in a dynamic flow environment, which may inform further research to understand the evolutionary advantage of the burst and cruise swimming styles in their natural habitat.

##  Supplemental Information

10.7717/peerj.4041/supp-1 Supplemental Information 1 Data of zebrafish locomotory patterns and fluid velocity and pressure field

## Additional Information and Declarations

### Data Availability

The following information was supplied regarding data availability: The raw data has been provided as a Supplemental File.
